# In Situ-Formed and Low-Temperature-Deposited Nb:TiO_2_ Compact-Mesoporous Layer for Hysteresis-Less Perovskite Solar Cells with High Performance

**DOI:** 10.1186/s11671-020-03366-1

**Published:** 2020-06-22

**Authors:** Miao Yu, Haoxuan Sun, Xiaona Huang, Yichao Yan, Wanli Zhang

**Affiliations:** 1 State Key Laboratory of Electronic Thin Films and Integrated Devices, University of Electronic Science and Technology of China, Chengdu, 611731 China; 2 Chengdu Technological University, Chengdu, 611730 China

**Keywords:** Perovskite solar cells, Titanium oxide, Low-temperature, Hysteresis

## Abstract

Recently, reported perovskite solar cells (PSCs) with high power conversion efficiency (PCE) are mostly based on mesoporous structures containing mesoporous titanium oxide (TiO_2_) which is the main factor to reduce the overall hysteresis. However, existing fabrication approaches for mesoporous TiO_2_ generally require a high-temperature annealing process. Moreover, there is still a long way to go for improvement in terms of increasing the electron conductivity and reducing the carrier recombination. Herein, a facile one-step, in situ, and low-temperature method was developed to prepare an Nb:TiO_2_ compact-mesoporous layer which served as both scaffold and electron transport layer (ETL) for PSCs. The Nb:TiO_2_ compact-mesoporous ETL-based PSCs exhibit suppressed hysteresis, which is attributed to the synergistic effect of the increased interface surface area caused by nano-pin morphology and the improved carrier transportation caused by Nb doping. Such a high-quality compact-mesoporous layer allows the PSCs assembled using optimized 2% Nb-doped TiO_2_ to achieve a remarkable PCE of 19.74%. This work promises an effective approach for creating hysteresis-less and high-efficiency PSCs based on compact-mesoporous structures with lower energy consumption and cost.

## Introduction

Organic-inorganic hybrid perovskites have been attracting great interest as promising light-absorbing materials owing to their large absorption coefficients, high carrier mobility, and ease of fabrication [1–5]. Perovskite-based solar cells, photodetectors, light-emitting diodes (LEDs), and even memory devices have been widely investigated and established [6–8]. Since the year 2009, the power conversion efficiency of perovskite solar cells (PSCs) has maintained rapid growth from 3.8% to over 25% under standard AM 1.5 illumination [9–12]. PSCs are generally fabricated with a mesoporous or planar structure [13–15]. To date, the reported PSCs with high power conversion efficiency (PCE) are typically based on a mesoporous structure containing an indispensable scaffold layer of metal oxide [16]. Titanium oxide (TiO_2_) has been commonly used as an electron transport layer. The typical mesoporous-type PSC presented by Seok has a structure of FTO/compact TiO_2_/mesoporous TiO_2_ and perovskite composite layer/perovskite upper layer/PTAA/Au [17]. It is generally known that the mesoporous TiO_2_ contributes most to reduce the overall hysteresis for mesoporous-type PSCs [18]. However, the fabrication of a mesoporous TiO_2_ layer often requires a high-temperature (> 450 °C) annealing treatment, leading to large energy consumption and limiting its application in flexible devices [19–21]. Compared with the mesoporous-type PSCs, planar-type PSCs can be fabricated using a low-temperature and low-cost process [22]. However, planar-type PSCs usually suffer from poor electron conductivity, severe charge recombination, and relatively lower crystallinity, resulting in low PCE with severe hysteresis behavior [23, 24].

Extensive efforts have been made to develop high-quality TiO_2_ electron transport layers (ETLs) with high electron mobility, such as through morphology optimization, surface modifications, and doping. In particular, a wide range of elements have been chosen to prepare TiO_2_ doping layers in PSCs, including Lithium (Li) [25, 26], Niobium (Nb) [27, 28], Platinum (Pt) [29], Sodium (Na) [30], Neodymium (Nd) [31], and Aluminum (Al) [32]. For instance, Liu et al. reported that the Li-doped TiO_2_ ETL was beneficial to the performance of the mesoporous-structure PSCs, especially for alleviating the hysteresis effect [26]. Liao et al. reported that the Pt-doped TiO_2_ ETL could improve the charge carrier extraction and injection efficiency in n-i-p PSCs [29]. Other ions such as Na, Nb, and transition metal ions [30, 31, 33–35] were used to modify surface or passivate defect of TiO_2_, contributing to reducing non-radiative recombination. Among these elements, Niobium metal (Nb) is a good candidate as a doping material for titanium oxide electron transport materials due to its similar radius with that of titanium. The results shown by Yin et al. demonstrated that Nb doping could make an improvement in both conductivity and mobility, simultaneously decrease the trap-state density of TiO_2_ ETLs for PSCs [27]. Despite these progresses, a relatively high-temperature (150 °C) treatment was mandatory and large hysteresis was still observed in PSCs based on Nb-doped TiO_2_. As is well known, current density-voltage (*J-V*) hysteresis is a critical issue that occurs frequently, especially in planar-structure PSC devices. Severe hysteresis can lead to instability of PSCs and degradation of PCE. For this reason, it is highly desired to develop a hysteresis-less PSC utilizing a simple and low-temperature method.

Here, we propose a facile one-step, in situ, and low-temperature (70 °C) strategy to develop hysteresis-less PSCs which contain a single Nb:TiO_2_ compact-mesoporous layer serving as both scaffold and ETL. The Nb:TiO_2_ layer contains a compact TiO_2_ bottom with nano-pin morphology on the surface, which can be utilized as a scaffold. The hysteresis index decreased significantly from 24.39% for the PSC based on bare TiO_2_ to 3.19% for that based on 2% Nb:TiO_2_ layer due to the collaborative effect of the increased interface surface area caused by nano-pin morphology on the surface and the improved carrier transportation rate because of the presence of Nb. The high-quality mesoporous layer allowed the PSCs to achieve remarkable PCE of 19.7%. This work promises an effective approach for achieving hysteresis-less and high-efficiency PSCs through scalable and inexpensive methods at low temperature.

## Methods

### Sample Preparation

First, the FTO substrates were successively put into acetone, alcohol, and deionized water to be ultrasonic cleaned of 30 min each. After that, the cleaned substrates were treated by a UV-ozone cleaner for 20 min and then placed in a petri dish. Second, liquid TiCl_4_ was dropped into deionized water under the temperature of 0 °C to prepare 0.1 M TiCl_4_ aqueous solution. Third, NbCl_5_ powder was put into the ethanol near the temperature of 0 °C to obtain 0.1 M NbCl_5_ ethanol solution. Then, X vol.% NbCl_5_ ethanol solution and (100-X) vol.% TiCl_4_ aqueous solution were dropped onto the surface of FTO substrates sequentially inside the petri dish. After hydrothermal reacting at 70 °C for 60 min, the Nb:TiO_2_ nano-pin feature was formed on the FTO substrates.

The perovskite absorption layer was deposited with the dynamic two-step spin-coating method [36]. First, the PbI_2_ precursor solution was obtained by adding 0.462 g PbI_2_ into 1 mL DMF. Meanwhile, the CH_3_NH_3_I (MAI) precursor solution was obtained by adding 0.1 g MAI into 2 mL isopropanol (99.5%, Aladdin). Second, 55 μL PbI_2_ precursor solution was spun onto the as-prepared Nb:TiO_2_ ETL film at 3000 rpm for 10 s. At this moment, 55 μL MAI precursor solution was dropped onto the sample immediately, and spinning was continued for 20 s. Finally, the whole film was annealed at 150 °C for 15 min.

The HTL precursor was obtained by stirring 1 mL chlorobenzene solution, which contained 72.3 mg Spiro-OMeTAD, 28 μL 4-tert-butylpyridine, and 17 μL Li-TFSI solution (520 mg mL^−1^). The precursor was spin-coated onto perovskite film at 2000 rpm for 30 s. Then, the Spiro-OMeTAD HTL with a thickness of around 250 nm was obtained.

### Characterization Methods

A field-emission scanning electron microscope (FE-SEM, SU8010, Hitachi) was carried out to study the morphologies of the samples. The absorption spectra were recorded with a UV-vis spectrophotometer (Shimadzu, UV-3600). Electrochemical impedance spectroscopy (EIS) was employed to understand the carrier transportation process by an electrochemical workstation (Autolab, PGSTAT 302 N). The current density-voltage (*J-V*) measurement was recorded using a digital source (Keithley 2400) with the assistance of the solar simulator (ABET Technologies, SUN 3000).

## Results and Discussion

**Fig. 1:**
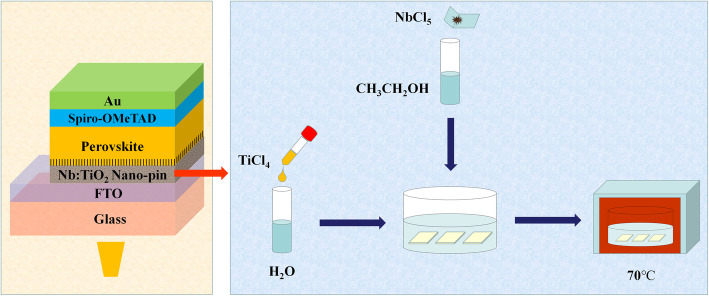
Schematic of PSC structure and Nb:TiO_2_ synthesis procedure

A schematic of the PSC structure and the Nb:TiO_2_ synthesis procedure is shown in Fig. 1. First, the cleaned FTO substrates were faced up placed in a petri dish. Second, 1 mL NbCl_5_ ethanol solution and 49 mL TiCl_4_ aqueous solution were poured onto the FTO substrates in the dish sequentially. Third, the dish was transferred into an oven and hydrothermal reacted at 70 °C for 1 h. Finally, the TiO_2_ layer with nano-pin morphology and 2% Nb doping ratio was formed on the FTO substrates. For the preparation of the control TiO_2_ layer, only TiCl_4_ aqueous solution (without NbCl_5_ ethanol solution) was dropped into the dish containing FTO substrates.

**Fig. 2:**
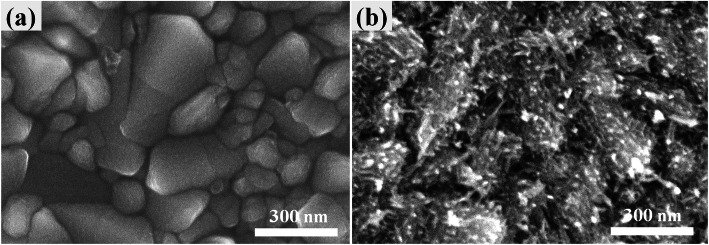
Top-view SEM images of **a** TiO_2_/FTO and **b** 2% Nb:TiO_2_/FTO

To understand the effect of Nb doping on the evolution of the TiO_2_ layer, the morphologies of the control TiO2 and Nb-doped TiO_2_ were investigated using scanning electron microscopy (SEM) which is shown in Fig. 2. The bare TiO_2_ exhibits a much smoother surface, which is a typical morphology of compact TiO_2_ layers in planar PSCs. However, 2% Nb-doped TiO_2_ shows a nano-pin texture distributed on the compact bottom. The length of the nano-pin was determined to be 50 ± 20 nm. This indicates that the Nb:TiO_2_ layer contains a compact TiO_2_ layer with a nano-pin morphology on the surface, which is regarded as a mesoporous layer. Therefore, this in situ formed Nb:TiO_2_ compact-mesoporous layer, which was obtained by a one-step process, actually serves as both a scaffold and an ETL in the PSC. The formation of nano-pin morphology resulted from the hydrothermal reacting with the assistance of NbCl_5_ ethanol solution.

**Fig. 3:**
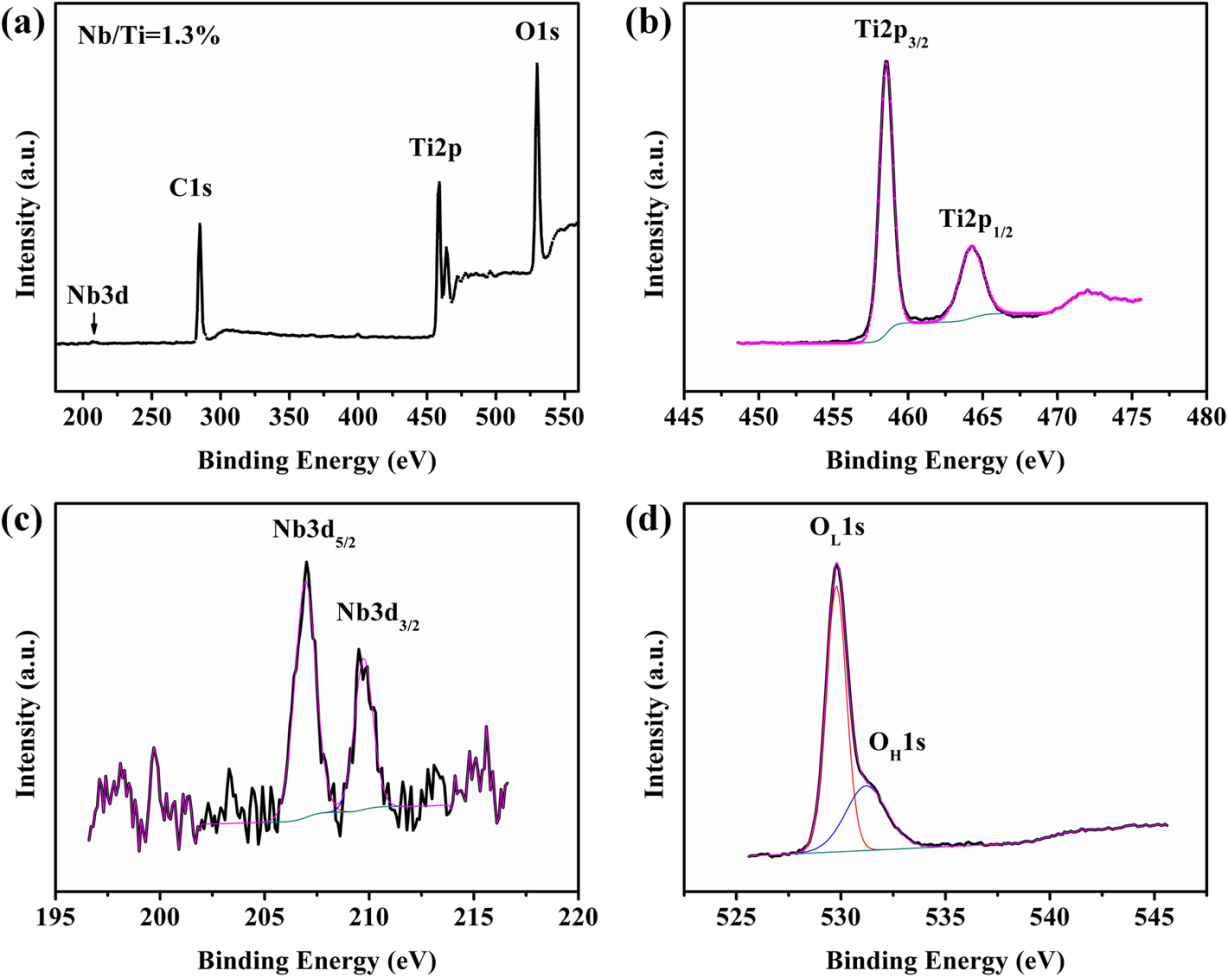
XPS spectra of 2% Nb:TiO2. **a** Survey, **b** Ti 2p, **c** Nb 3d, and **d** O 1s

The XPS spectra of 2% Nb:TiO_2_ film is shown in Fig. 3. Figure 3a shows the full scan spectra of the 2% Nb:TiO_2_ film. It is found that the atom ratio of Nb/Ti (1.3%) is closed to the element doping ratio of 2% in the precursor mixture. As shown in Fig. 3b, the Gaussian peaks located at 458 eV and 464 eV are corresponding to the binding energy of Ti 2p_3/2_ and Ti 2p_1/2_. Similarly, the Gaussian fitted lines of Nb^5+^ can be deconvolved into two individual peaks which are associated with Nb 3d_5/2_ and Nb 3d_3/2_, respectively, at the binding energy of 207 eV and 209 eV (Fig. 3c). The XPS spectra demonstrate the successful doping of Nb in the TiO_2_ film.

**Fig. 4:**
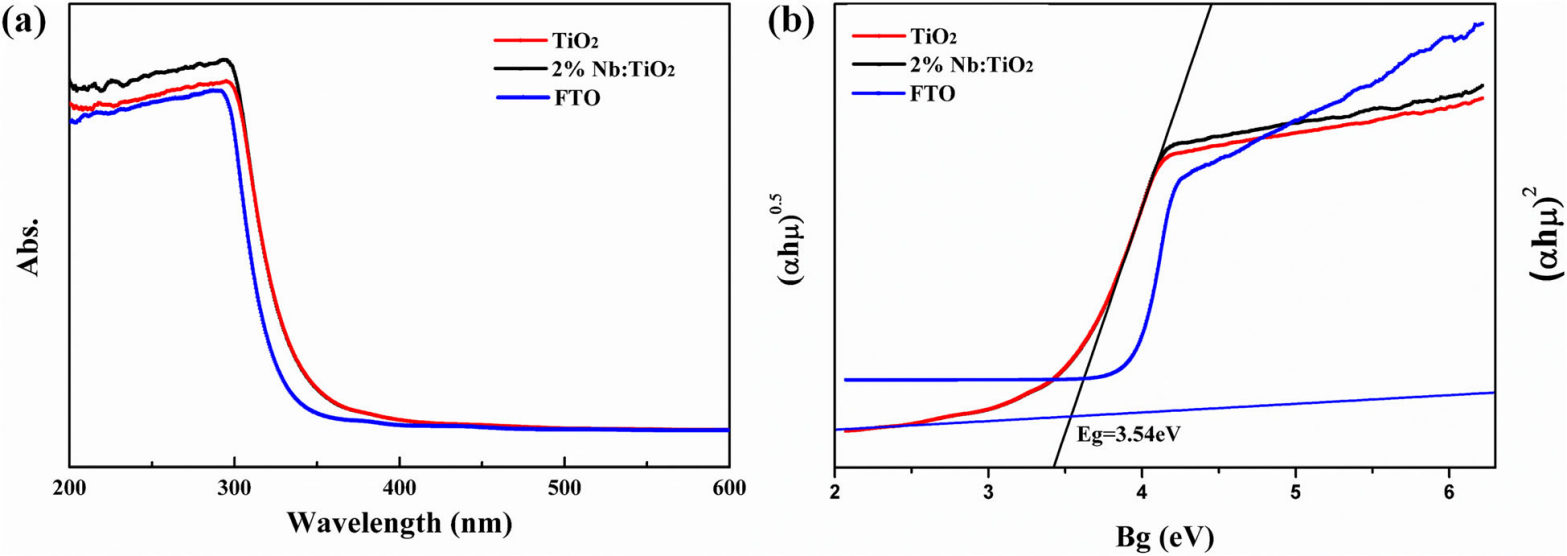
**a** The absorption spectra of the FTO substrate, TiO_2_/FTO, and 2% Nb:TiO_2_/FTO. **b** Tauc-plots of the FTO substrate, TiO_2_/FTO, and 2% Nb:TiO_2_/FTO

Figure 4a shows the absorption spectra of FTO, bare TiO_2_/FTO, and Nb-doped TiO_2_/FTO. Both bare TiO_2_ and Nb-doped TiO_2_ exhibit main absorption edge at the wavelength of 300–350 nm. The absorption curve of Nb-doped TiO_2_ almost overlaps that of bare TiO_2_. The energy bandgap (*E*_g_) can be calculated based on the absorption spectra using the Tauc equation, which is shown in Fig. 4b. The *E*_g_ is 4.05 eV for FTO and 3.5 eV for both bare TiO_2_ and Nb-doped TiO_2_. Therefore, it can be concluded that Nb doping has little influence on the absorption of TiO_2_. The transmittance is also not shifted during the Nb doping process as shown in Fig. S1.

Fig. S2 presents the SEM images of CH_3_NH_3_PbI_3_ perovskite films spin-coated on the bare TiO_2_ and Nb-doped TiO_2_ films. It is indicated that the perovskite films exhibit fewer pinholes and full surface coverage. Thanks to our previously developed non-substrate-selective dynamic two-step spin-coating strategy [36], the film uniformity and coverage can be better controlled. Besides, the average crystalline grain sizes of the perovskite films are very similar. Fig. S3 presents the absorption spectra of the perovskite films deposited on the bare TiO_2_ and Nb-doped TiO_2_ films. No obvious difference in absorption peak is observed between the perovskite films. These results suggest that the nano-pin morphology formation on the Nb-doped TiO_2_ compact-mesoporous layer could have little effect on the perovskite crystallization by dynamic two-step spin-coating strategy.

**Fig. 5:**
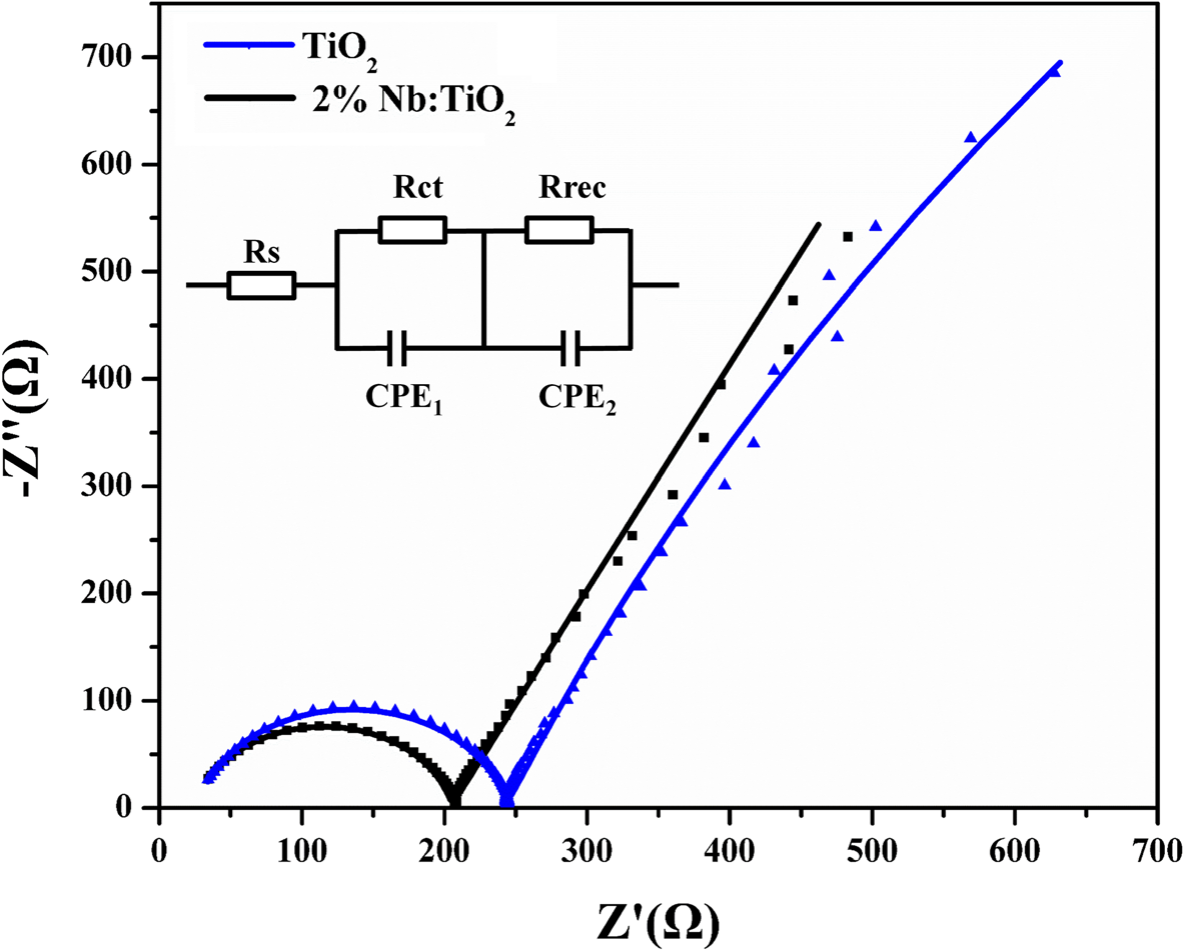
Nyquist plots of devices based on bare TiO_2_ and 2% Nb-doped TiO_2_ layers

To understand the carrier transportation crossing the ETL/perovskite interfaces, the electrical impedance spectroscopy (EIS) was employed. PSCs were fabricated with the structure of FTO/TiO_2_/perovskite film/Spiro-OMeTAD/Au. Figure 5 shows the Nyquist plots of PSCs based on bare TiO_2_ and 2% Nb:TiO_2_ layers, and the corresponding equivalent circuit model is shown in the inset. The parameters of EIS were listed in Supplementary Table S1. It is known that the EIS contains two circular arcs [37]. The high-frequency component is attributed to the charge transport resistance (*R*_ct_), and the low-frequency component is mainly related to the recombination resistance (*R*_rec_) [38]. In this comparison, everything but the perovskite/ETL interface was identical. Thus, only the Nb doping process should be responsible for the resistance (*R*_ct_ and *R*_rec_) variation. Compared to the bare TiO_2_ device, the Nb:TiO_2_ device exhibits smaller *R*_ct_ and larger *R*_rec_. The small *R*_ct_ contributes to more efficient electron extraction, and the large *R*_rec_ proves lower charge recombination. These results confirm that the Nb:TiO_2_-based compact-mesoporous layer is an effective ETL for both charge transportation improving and carrier recombination rate reducing.

**Fig. 6:**
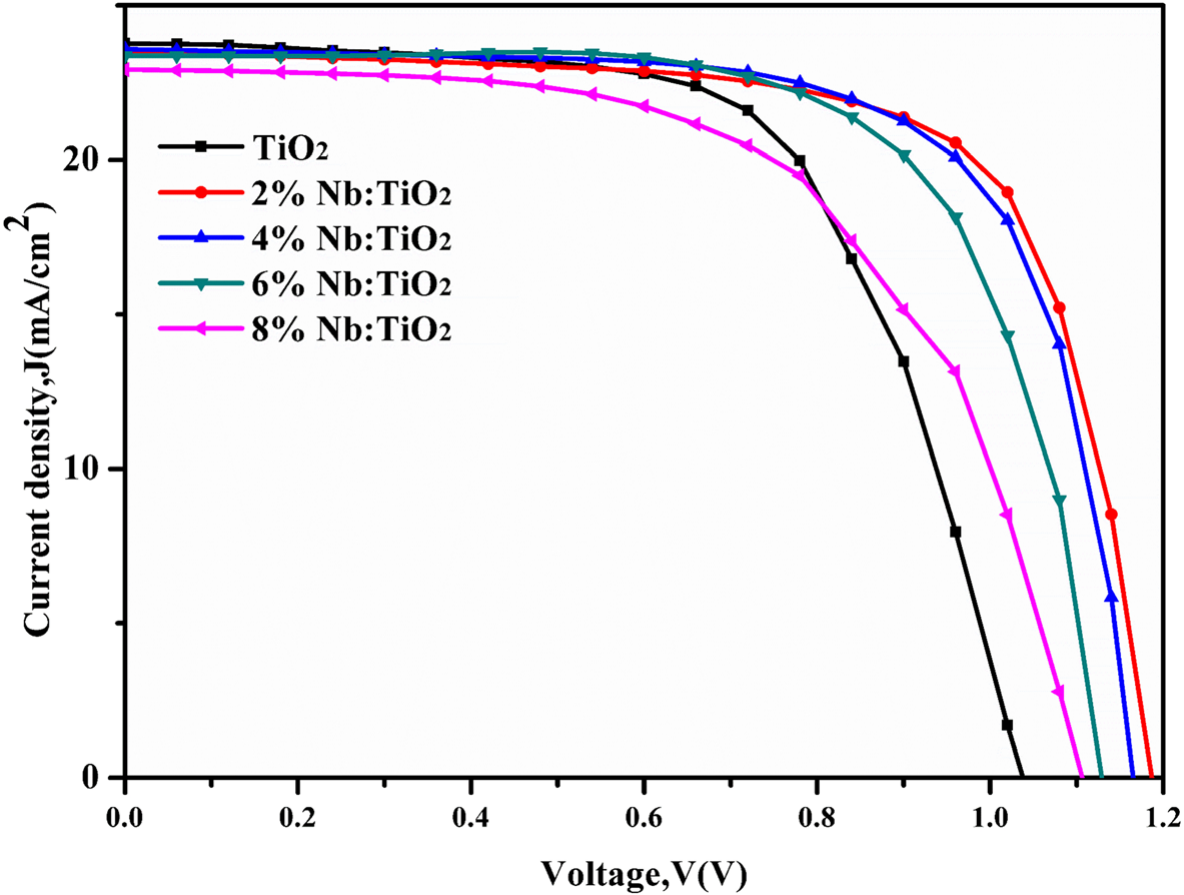
*J-V* curves of PSCs based on different Nb doping concentrations

As shown in Fig. 6, the dependence of the PCE of PSCs on the Nb doping contents was investigated. The detail parameters for PSCs with different Nb doping concentrations varying from 0 to 8% was shown in Table 1. It is found that the doping ratio affects the open-circuit voltage (*V*_oc_) and fill factor (FF), which were first increased and then decreased with increasing Nb doping. The device with a 2% Nb-doped TiO_2_ layer exhibits the highest *V*_oc_ of 1.19 eV, *J*_sc_ of 23.52 mA/cm^2^, and FF of 70.74%, leading to a PCE as high as 19.74% for the champion devices. Thanks to better carrier transportation, all parameters show notable improvement. However, superfluous doping would strengthen the carrier scattering and lead to poor mobility. The incremental recombination will weaken the carrier transport improvement and eventually harm the PCE.

**Table 1:** Performance parameters of PSCs based on different Nb doping concentrations

Solar cells	***V***_**oc**_ (V)	***J***_**sc**_ (mA cm^**−2**^)	FF (%)	PCE (%)
0% Nb	1.04	23.80	63.56	15.70
2% Nb	1.19	23.52	70.74	19.74
4% Nb	1.17	23.58	70.28	19.31
6% Nb	1.12	23.36	68.88	18.17
8% Nb	1.11	22.93	59.94	15.21

**Fig. 7:**
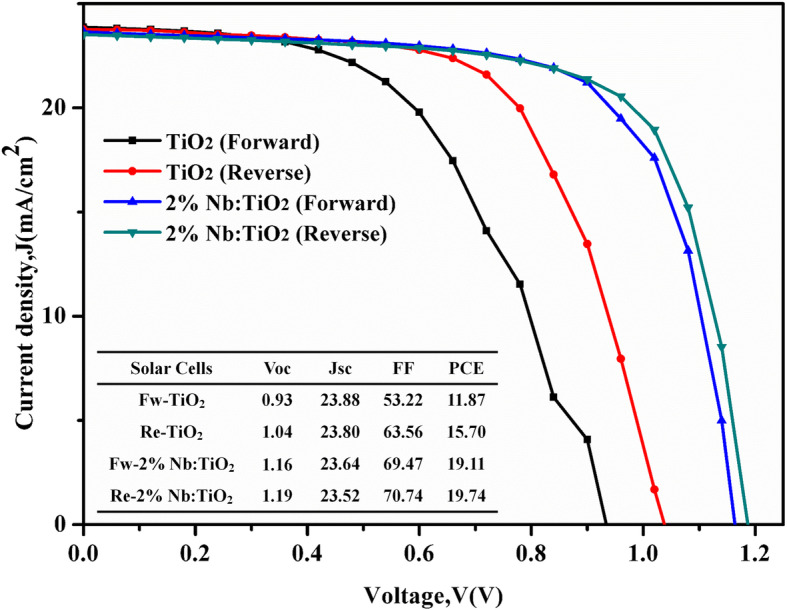
The *J-V* hysteresis behavior of the PSCs based on bare TiO_2_ and 2% Nb:TiO_2_ layer under AM 1.5 illumination

The measured *J-V* curves of the control and champion device are shown in Fig. 7. It is well known that *J-V* hysteresis behavior often occurs, especially in planar-structure PSC devices. In this work, the hysteresis of *J-V* curves of bare compact TiO_2_-based PSC and 2% Nb:TiO_2_ compact-mesoporous layer-based PSC were examined. The hysteresis index, (PCE of reverse scan − PCE of forward scan)/PCE of reverse scan [30], reduced markedly from 24.39% for the PSC based on bare compact TiO_2_ to 3.19% for the PSC based on 2% Nb-doped TiO_2_ layer. It is well known that PSCs based on a mesoporous TiO_2_ layer can collect electrons and effectively achieve a balance between the hole flux and electron flux due to its larger surface area, thereby exhibiting less hysteresis [17]. The hysteresis suppression of the Nb-doped TiO_2_-based device is motivated by the conductance increasing and the nano-pin morphology forming. Charge accumulation caused by interfacial capacitance at the ETL/perovskite interface would be reduced and result in hysteresis-less character.

## Conclusion

We have developed a facile one-step, in situ, and low-temperature approach to achieve an Nb:TiO_2_ compact-mesoporous layer that serves as both scaffold and ETL for PSCs. As a result, PSCs based on 2% Nb-doped TiO_2_ can exhibit a remarkable PCE of 19.74%, which is dramatically higher than that of the controlled TiO_2_-based device. The Nb:TiO_2_ layer contains a compact TiO_2_ bottom with nano-pin morphology on the surface, which can be utilized as a mesoporous layer. Due to the collaborative effect of a large interface surface area and improved carrier transportation rate, the hysteresis of the *J-V* curve is markedly reduced, with the hysteresis index decreasing significantly from 24.39 to 3.19%. This work promises an effective approach for achieving hysteresis-less and high-efficiency PSCs through a well-designed scalable and cost-efficiency hydrothermal method at low temperature.

## Additional File

**Additional file 1: Figure S1.** The light permeation comparison of TiO_2_ and Nb:TiO_2_ based ETL. **Figure S2.** SEM images of (a) perovskite deposited on the pure TiO_2_ and (b) perovskite deposited on the TiO_2_ layer doped with 2% Nb. **Figure S3.** Absorbance spectra of perovskite film deposited on pure TiO_2_ and 2%Nb:TiO_2_ layer. **Table S1.** Parameters employed for the fitting of the impedance spectra of devices based on the pure TiO_2_ and 2% Nb:TiO_2_

## Data Availability

The authors declare that the materials and data are available to the readers, and all conclusions made in this manuscript are based on the data which are all presented and shown in this paper.

## Abbreviations

PSCs: Perovskite solar cells
PCE: Power conversion efficiency
TiO_2_: Titanium oxide
ETL: Electron transport layer
SEM: Scanning electron microscope
EIS: Electrochemical impedance spectroscopy
*B*_g_: Bandgap
*E*_g_: Energy bandgap
*V*_oc_: Open-circuit voltage
FF: Fill factor
*J*_*sc*_: Short circuit current density
