# Supplementary material for: In Situ-Formed and Low-Temperature-Deposited Nb:TiO2 Compact-Mesoporous Layer for Hysteresis-Less Perovskite Solar Cells with High Performance
Source: Nanoscale Res Lett. 2020 Jun 22;15:135. doi: 10.1186/s11671-020-03366-1 (PMC7310023; doi:10.1186/s11671-020-03366-1)
Supplement: Supplementary file 1 — Additional file 1: Figure S1. The light permeation comparison of TiO2 and Nb:TiO2 based ETL. Figure S2. SEM images of (a) perovskite deposited on the pure TiO2 and (b) perovskite deposited on the TiO2 layer doped with 2% Nb. Figure S3. Absorbance spectra of perovskite film deposited on pure TiO2 and 2%Nb:TiO2 layer. Table S1. Parameters employed for the fitting of the impedance spectra of devices based on the pure TiO2 and 2% Nb:TiO2 [file 11671_2020_3366_MOESM1_ESM.docx]

In-situ formed and low-temperature deposited Nb: TiO_2_ compact-mesoporous layer for hysteresis-less perovskite solar cells with high performance

Miao Yu^1^, Haoxuan Sun^1^, Xiaona Huang^2^, Yichao Yan^1,*^, Wanli Zhang^1^

(^1^State Key Laboratory of Electronic Thin Films and Integrated Devices, University of Electronic Science and Technology of China, Chengdu, 611731, China；

^2^Chengdu Technological University, Chengdu, 611730, China)

Miao Yu: yumiao@uestc.edu.cn

Haoxuan Sun: 2577187880@qq.com

Xiaonan Huang: 504251083@qq.com

Yichao Yan: yanyichao@uestc.edu.cn

Wanli Zhang: wlzhang@uestc.edu.cn


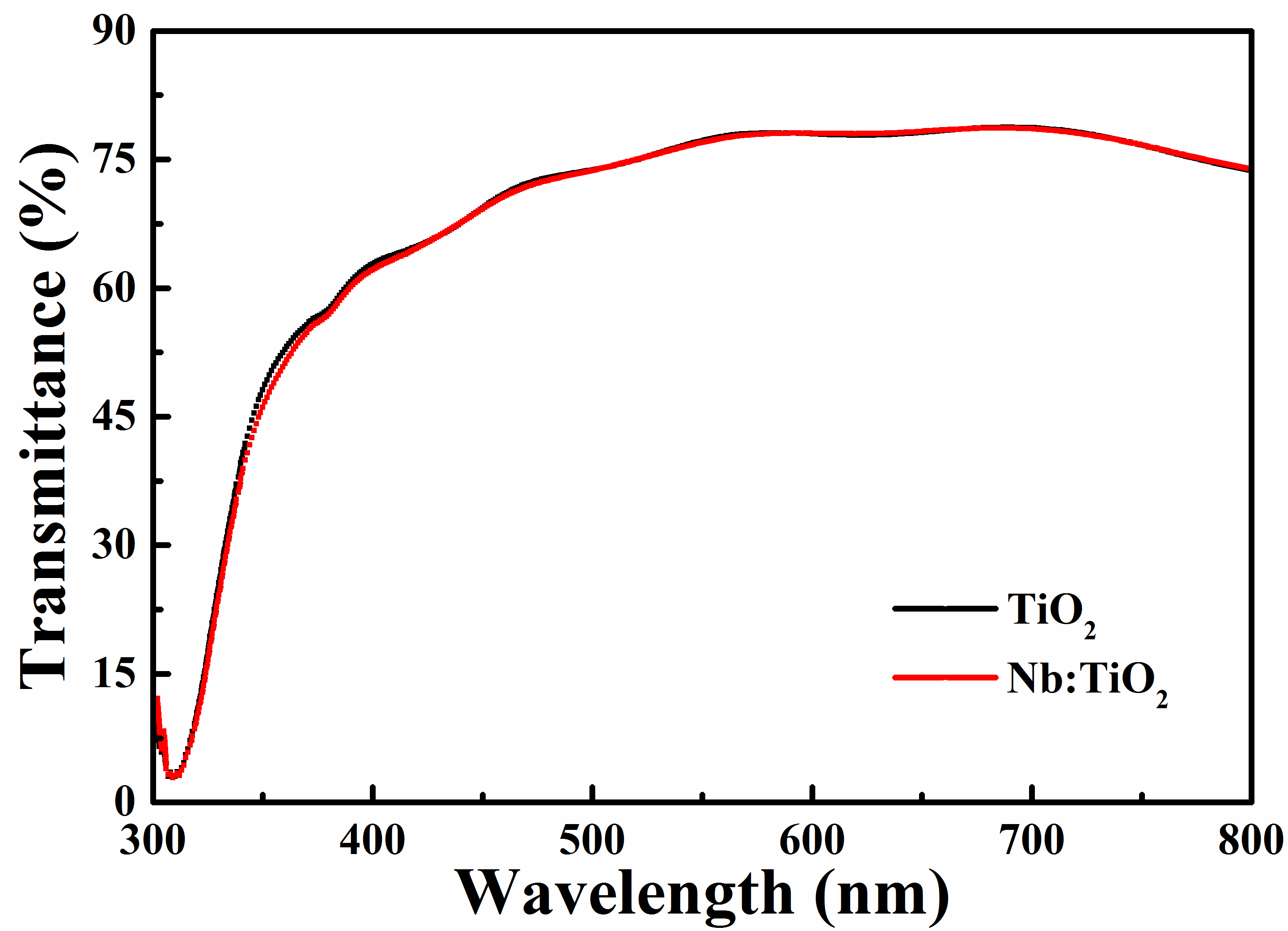


Fig. S1 The light permeation comparison of TiO_2_ and Nb:TiO_2_ based ETL


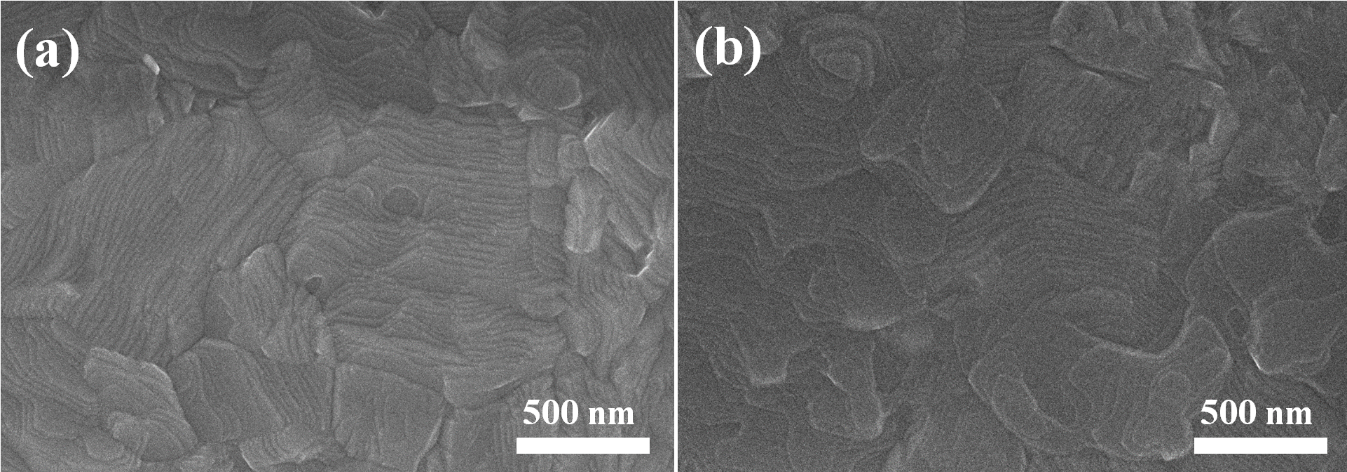


Fig. S2 SEM images of (a) perovskite deposited on the pure TiO_2_ and (b) perovskite deposited on the TiO_2_ layer doped with 2% Nb





Fig. S3 Absorbance spectra of perovskite film deposited on pure TiO_2_ and 2%Nb:TiO_2_ layer

| **Device** | ***R_s_* (Ω)** | ***R_ct_* (Ω)** | ***R_rec_* (kΩ)** |
| --- | --- | --- | --- |
| TiO_2_ | 27.1 | 216 | 5.23 |
| 2% Nb:TiO_2_ | 26.2 | 178 | 119 |

**Table S1** Parameters employed for the fitting of the impedance spectra of devices based on the pure TiO_2_ and 2%Nb:TiO_2_
